# Provision and Supervision of Food and Protein Substitute in School for Children with PKU: Parent Experiences

**DOI:** 10.3390/nu13113863

**Published:** 2021-10-28

**Authors:** Hannah Jones, Alex Pinto, Sharon Evans, Suzanne Ford, Mike O’Driscoll, Sharon Buckley, Catherine Ashmore, Anne Daly, Anita MacDonald

**Affiliations:** 1 Faculty of Health, Education & Life Sciences, Birmingham City University: City South Campus, Westbourne Road, Edgbaston, Birmingham B15 3TN, UK; Hannah.Jones12@mail.bcu.ac.uk; 2 Birmingham Women’s and Children’s NHS Foundation Trust, Steelhouse Lane, Birmingham B4 6NH, UK; alex.pinto@nhs.net (A.P.); sharon.morris6@nhs.net (S.E.); catherine.ashmore@nhs.net (C.A.); a.daly3@nhs.net (A.D.); 3 National Society for Phenylketonuria, Sheffield S12 9ET, UK; suzanne.ford@nspku.org; 4 North Bristol NHS Trust, Southmead Road, Bristol BS10 5NB, UK; 5 School of Health and Education, Middlesex University, Room WG41A (Williams Building), The Burroughs Hendon, London NW4 4BT, UK; m.odriscoll@mdx.ac.uk; 6 Department of Psychology, Faculty of Health, Psychology and Social Care, Manchester Campus, Manchester Metropolitan University, 53 Bonsall Street, Manchester M15 6GX, UK; sharon.j.buckley@stu.mmu.ac.uk

**Keywords:** PKU, food, protein substitute, school, IHCP, parent/caregiver experiences

## Abstract

Children spend a substantial part of their childhood in school, so provision of dietary care and inclusion of children with phenylketonuria (PKU) in this setting is essential. There are no reports describing the dietary support children with PKU receive whilst at school. The aim of this cross-sectional study was to explore the experiences of the dietary management of children with PKU in schools across the UK. Data was collected using an online survey completed by parents/caregivers of children with PKU. Of 159 questionnaire responses, 92% (*n* = 146) of children attended state school, 6% (*n* = 10) private school and 2% (*n* = 3) other. Fourteen per cent (*n* = 21/154) were at nursery/preschool, 51% (*n* = 79/154) primary and 35% (*n* = 54/154) secondary school. Sixty-one per cent (*n* = 97/159) said their child did not have school meals, with some catering services refusing to provide suitable food and some parents distrusting the school meals service. Sixty-one per cent of children had an individual health care plan (IHCP) (*n* = 95/155). Children were commonly unsupervised at lunchtime (40%, *n* = 63/159), with snacks (46%, *n* = 71/155) and protein substitute (30%, *n* = 47/157), with significantly less supervision in secondary than primary school (*p* < 0.001). An IHCP was significantly associated with improved supervision of food and protein substitute administration (*p* < 0.01), and better communication between parents/caregivers and the school team (*p* < 0.05). Children commonly accessed non-permitted foods in school. Therefore, parents/caregivers described important issues concerning the school provision of low phenylalanine food and protein substitute. Every child should have an IHCP which details their dietary needs and how these will be met safely and discreetly. It is imperative that children with PKU are supported in school.

## 1. Introduction

In the UK, it is estimated there are approximately 800 children with phenylketonuria (PKU) aged 5 to 16 years [1]; they are expected to attain normal educational achievement and attend mainstream school. Children with classical PKU are treated with a phenylalanine restricted diet only; if they have mild PKU they may be treated with an adjunct therapy, sapropterin. Children with classical PKU usually tolerate < 80% of usual natural protein intake and treatment includes: avoidance of high protein foods, strict measurement and limited intake of moderate protein containing foods, inclusion of special low protein foods (SLPF’s) and supplementation with a low phenylalanine protein substitute [2]. Most children will be expected to eat at least one meal and take one dose of protein substitute at school. It is essential that there is safe provision and supervision of dietary treatment with appropriate adjustments that integrates the medical needs of a child with PKU into school life. 

Section 100 of the UK Children and Families Act 2014, updated in 2015, states that schools in the UK have a duty to support pupils with medical conditions [3,4]. This act mandates that children with PKU are properly supported, enabling them to have a full and active role in school, remain healthy and achieve their academic potential. It states that school leaders should consult health and social care professionals, pupils, and parents so that the needs of children with medical conditions are accurately understood and effectively met. Schools have a duty to ensure that all relevant staff are trained to provide the support that pupils’ need, and that policies, plans, procedures, and systems are implemented. Although not mandatory, each school should have policies to ensure all relevant staff are aware of the child’s condition; that there are cover arrangements in case of staff absences or staff turnover, and that risk assessments are conducted for school visits, holidays, and other activities outside the normal timetable. Failure to make reasonable adjustment for a child with a disability is considered discrimination under the UK Equality Act 2010 [5]. 

Ideally each child with PKU should have an individual health care plan (IHCP) although these are not obligatory by law [4]. These should be developed in partnership between the school, parents, pupils, and relevant healthcare professionals who can advise on individual medical care needs. An IHCP should ensure that schools know how to support children with PKU effectively by providing clarity about what needs to be done, when and by whom. They should be reviewed at least annually or earlier if health care needs change. School governing bodies should ensure that their schools have policies and appoint staff who are responsible for managing IHCP’s. 

In addition, in UK state-funded schools, every child in reception, year 1 and 2 (children aged 4–7 years) are entitled to a free school lunch [6]. They should have access to a healthy, balanced diet and it is recommended that they have at least one hot meal provided every day. Food and drinks provided by school must comply with certain nutritional standards [7] and reasonable adjustment should be made for children on special diets. The Education Act 1996 requires maintained schools and academies to provide free school meals to disadvantaged pupils aged between 5 to 16 years, with 20.8% of children in England (2020/2021) being entitled to this service [8]. 

Dietary treatment is expected to have both a physiological and psychological impact on the lives of young people with PKU in school. Whilst consumption of non-permitted foods and poor adherence to protein substitute will lead to elevated blood phenylalanine and neurological dysfunction, teacher/peer insensitivity and exclusion may have an enduring impact on a child’s mental health, and attitude and acceptance of PKU. There are no studies examining care provision in school and the opinions and experiences of parents of school children with PKU are unknown. The aim of this study was to explore the views and experiences of parents/caregivers of children with PKU in school and nursery. Additionally, the care of children with and without an IHCP was also studied. 

## 2. Materials and Methods

### 2.1. Study Design

This was a cross-sectional study using an online survey that collected both qualitative and quantitative data from UK parents of children aged 3 to 16 y with PKU attending school or nursery. Non-UK respondents were excluded. 

The questionnaire was built in the Online Surveys platform (https://www.onlinesurveys.ac.uk, accessed on 28 October 2021) to gather quantitative data. This was placed on the UK National Society for Phenylketonuria (NSPKU) website, with additional promotion on the NSPKU Twitter, Instagram and Facebook. The survey was open for five months, from 20 March until 20 August 2020.

### 2.2. Questionnaire

The non-validated questionnaire contained 22 questions: *n* = 17 multiple choice (with *n* = 14 inviting additional comments), *n* = 3 multiple responses, *n* = 1 Likert scale and *n* = 1 open ended questions (Supplementary Material). 

The questionnaire was developed collaboratively by dietitians with expert practical and scientific knowledge of PKU (AP, SE, AM), a colleague from the NSPKU (SF), a researcher (MO) and a student dietitian from Birmingham City University (HJ). It was reviewed amongst colleagues and lay people to ensure its readability and then amended according to feedback.

### 2.3. Data Collected

The questionnaire was divided into four sections. Information collected included: the age of the child, type of school, school year group, the availability of an IHCP, administration of protein substitute in school, provision and acceptance of lunches provided by school catering services, information about the suitability of school lunches, school staff training and supervision of food and protein substitute. All data that was collected was based on the parents own perception or knowledge about the quality of the care and support provided by the nursery or school. 

### 2.4. Statistics

Quantitative data analysis (inferential and descriptive statistics) was carried out with the Statistical Package for the Social Sciences (SPSS) version 25 (SPSS Inc., Chicago, IL, USA). Multiple response questions were analysed with descriptive statistics only. Statistical significance was set at *p* < 0.05. 

Qualitative data analyses of 14 open-ended responses were carried out in NVIVO v 12 PRO. The whole survey dataset was imported into NVIVO, so that coding of open-ended responses could be broken down by attributes of survey questions. All open-ended question responses were analysed thematically.

### 2.5. Ethics

Ethical approval was obtained from the Birmingham City University ethics committee prior to commencement of the study (Jones/5042/R(A)/2020/Mar/HELS FAEC - Provision of school food for children with PKU: A parent’s perspective. Approved 19/3/2020). At the beginning of the online questionnaire, respondents gave consent, and it was emphasized that questionnaire completion was voluntary. Potential respondents were advised that data from the survey may be published in an anonymized form. If names of schools or hospitals were mentioned in verbatim abstracts these were removed from results presented in this manuscript.

## 3. Results

There were 159 responses. The number of respondents who answered each question was variable (as not all questions were applicable to each respondent). All respondents were parents/caregivers of children with PKU. A description of the school type, school age group and provision of IHCP for children is given in Table 1. 

### 3.1. Uptake of School Meals

Uptake of school lunches and entitlement to free school meals is given in Table 2. Most parents/caregivers (61%, *n* = 96/157) said their children were not eating meals provided by the school catering service.

Sixty-two per cent (*n* = 73/117) of parents/caregivers said that they would like their child to have school lunches more often. Only 52% (*n* = 29/56) utilized their free school lunch entitlement. Of those with free school meal entitlement, 41% were eating school lunches 4–5 times a week compared to 16% of those without the entitlement (Pearson Chi-Square test *p* = 0.05). Of the children eating school lunches, 76% (*n* = 48/63) of parents were satisfied with the school lunch service.

Respondents were asked in two open-ended questions, about barriers to accessing school meals more frequently. The main themes which emerged were: school refusing to cater for children with PKU, limited food choice offered by school, child or parent preferring packed lunch, parents did not trust school to prepare appropriate food for their child with PKU, parents were more in control of what their child eats with packed lunches, and children refuse school meals because they openly advertise that they are different. Some parents described how the school or school catering were unwilling or reluctant to cater for children with PKU, particularly in secondary school. They described the inflexibility of catering services, how some parents had to supplement the school lunch with food prepared at home, and exclusion from special occasion meals such as Christmas dinner.

Parents/caregivers verbatim quotes:

- “*The school use an outside catering company who were not prepared to cook any food that was not sourced by them.*”
- “*School refused to provide school lunches due to health and safety.*”
- “*Not comfortable with someone else having control of portions in case they aren’t weighed properly, or wrong foods given by mistake.*”
- “*Tried school lunches. Blood phenylalanine levels went too high. Child was not supervised.*”

### 3.2. Food Included in School Lunch Service

The type of school meal plans and variety of low protein foods given are outlined in Table 3.

Parents usually supplied the SLPF’s such as pasta and bread which they obtained on prescription; the school usually provided low protein/vegan cheese and ‘fishless’ fingers purchased from wholesalers. Some parents said the school ‘do not provide anything.’ Children with an IHCP (68%, *n* = 25/37) were much more likely than those without IHCP (50%, *n* = 7/14) to have alternative meals prepared but the difference was not statistically significant (Pearson Chi Square test *p* > 0.05). Children in private school were more likely to have a separate meal prepared (100%, *n* = 5/5) compared with 58% (*n* = 26/45) of state schools, but the difference did not reach statistical difference due to the small numbers of children in private school. There were no clear differences related to the school year of the child.

Fifty-nine percent (*n* = 37/63) said catering staff measured or weighed protein exchange foods (e.g., mashed potato or peas) and 2% (*n* = 1/63) were unaware if foods were measured. Some parents commented that it was unnecessary for the school to weigh protein exchanges because they either provided the food pre-measured, the main meal did not contain protein exchanges, or they did not ask the school catering to weigh exchange foods.

Weighing and measuring of food protein exchanges was most common (80%, *n* = 12/15) in nursery/reception school compared to other school age groups (57%, *n* = 24/42) [Pearson Chi-Square test, *p* = 0.014]. Parents/caregivers were asked to score satisfaction with the school meal service on a scale of 1 (extremely dissatisfied) to 5 (extremely satisfied). They gave a higher satisfaction score (median 5) when the school measured/weighed protein exchanges compared with scoring for schools who did not weigh/measure protein exchanges (median 4) (Mann–Whitney U test, *p* = 0.003)

There were some parent comments about the quality, variety and presentation of food provided by the school catering service.

Parents/caregivers verbatim quotes:

- “*The dinners came from another school and the presentation when they arrived was not that appetising.*”
- “*Would like a wider choice of salads being provided and more attractive fruit at lunches.*”
- “*Some of the protein exchanges were noted wrongly and also weighed out incorrectly.*”

### 3.3. Training and Knowledge about PKU and Diet

Parents/caregivers said that only 47% (*n* = 74/159) of their child’s class teachers and 54% (*n* = 33/61) of catering staff (for those receiving school meals) had received PKU training from a health professional. Of the teachers and catering team who had received training, 82% (*n* = 58/71) of teachers and 85% (*n* = 35/41) of the catering team received training in the previous 2 years. The training was mainly delivered by the child’s dietitian.

### 3.4. Supervision of Food in School

Children were commonly unsupervised at lunchtime (43%, *n* = 66/154) or snack time (48%, *n* = 74/155). Lack of meal supervision was significantly more common in secondary schools (61%, *n* = 33/54) than in primary schools (27%, *n* = 21/79) (Pearson Chi-Squared test *p* < 0.001).

Those without an IHCP (40%, *n* = 59/148) were more commonly unsupervised at school at meal and snack time (60%, *n* = 32/53) compared to those who had a plan (28%, *n* = 27/95) (Pearson Chi-Square test *p* < 0.01). Of the children supervised at lunchtime, school lunchtime supervisors most commonly did this task (27%, *n* = 24/88), whereas snacks were mainly supervised by teaching assistants (30%, *n* = 24/81). 

### 3.5. Feedback about Food Eaten in School

Only 36% (*n* = 57/157) of parents/caregivers said they received feedback about what their children eat in school. Feedback was more common for children with an IHCP in a state school compared with children without one (Pearson Chi-square test *p* < 0.05); and more common for children in nursery/reception and primary school (year 1 to 3) (64%, *n* = 27/42) than in secondary school (15% *n* = 8/53) (Pearson Chi-square test *p* < 0.001). It was marginally more common in private school (40%, *n* = 4/10) compared to state school (35%, 51/144) [Pearson Chi-square test *p* > 0.05].

When feedback was received, 56% (*n* = 32/57) of parents/caregivers received a written record of food eaten, 25% (*n* = 14/57) verbal feedback and 11% (*n* = 6/57) photographs of food eaten via online systems. Nine per cent (*n* = 5/57) received feedback in ‘other’ forms such as: lunch wrappers and uneaten food being left in the bag (as evidence of what has been eaten), the online system for monitoring school meal purchases, messages in a schoolbook/homework book, and an email or telephone call from the school.

### 3.6. Incidents of Eating Foods at School That Were Not Permitted

Parents reported 53 incidents of incorrect foods being given accidentally/purposely to children in school in the previous 6 months. Forty per cent (*n* = 21/53) of parents/caregivers said that it had happened once; 19% (*n* = 10/53) said 2 to 3 times, 8% (*n* = 4/53) said 4 to 5 times and 34% (*n* = 18/53) said that it had happened more than five times. Respondents were asked to describe incidents of their child eating non permitted food at school, and these responses (*n* = 39) were thematically analysed. The main themes describing incidents were associated with staff errors (*n* = 4), other children sharing inappropriate foods (*n* = 11), child choosing inappropriate foods (*n* = 5) and trying to fit in with others (*n* = 4). Two parents mentioned that they felt it was much harder for the school to supervise the child’s eating once they were in secondary school.

Parents/caregivers verbatim quotes:

- “*He was given an incorrect lunch when the school cook was on holiday.*”
- “*She asked her friend to buy her foods like toast and chocolate from the tuck bar each morning.*”
- “*I’ve saw on ‘parent pay’ that he purchased baked goods such as flapjacks and cakes.*”
- “*Because she felt left out so she was going into the canteen on chip day and buying double her amount.*”

Secondary school children were much more likely to have eaten foods which were not permitted as part of a low phenylalanine diet (45% (*n* = 10/22) of secondary school children (year 10 to 11) compared with 26% (*n* = 9/35) of primary school children (Year 1 to 3) but the differences were not statistically significant (Pearson Chi-square test *p* > 0.05). 

Two-thirds (66%, *n* = 35/53) of parents/caregivers said that they did not feel adequately informed about food incidents. Parents/caregivers were much more likely to say that they felt adequately informed of the incident if children were in nursery/reception (60%, *n* = 3/5) and primary school (years 4-6) (58%, *n* = 7/12) [Pearson Chi-square test *p* > 0.05]. Respondents were asked (open-ended question) to comment about the communication they received from the school staff about food incidents. The main common themes from the 25 responses were: informed by child (*n* = 7), staff were slow or late in informing us (*n* = 4), should be greater staff understanding or awareness (*n* = 4), and staff don’t care (*n* = 3).

Parents/caregivers verbatim quotes:

- “*Well, they were not sure what she really ate. My daughter told me what she ate and at the end they confirmed this.*”
- “*I was not informed. Being in a secondary school the PKU diet is hard to monitor for all staff and they are not able to monitor my son’s actions.*”
- “*The teachers don’t understand the condition so she is left to get on with it.*”

### 3.7. School Strategies to Prevent Children Being Given the Incorrect Foods at School

The parents of nursery/reception and primary school (years 1–3) children were much more likely to state that there were strategies in place to prevent incorrect food being eaten at school compared with older children with PKU (Pearson Chi-Square test *p* < 0.001). Thirty-eight (*n* = 60/158) of respondents said there were no procedures in place to prevent such incidents reoccurring. However, parents gave many examples of strategies used by the school staff to try and ensure children were given the correct food Table 4.

### 3.8. Exclusion: Feeling and Looking Different in School

Thematic analysis of general comments received about provision of food in school showed that parents/caregivers were concerned that their child was either excluded from activities/school events because of PKU or that they looked different from others in school.

Parents/caregivers verbatim quotes:

- “*My teenage son does not want attention brought to his PKU. Refuses to have special food at school or anyone know about his PKU.*”
- “*My child does not want to stay for lunch as she only likes to eat chips and the school would have to measure them out. This would lead to others asking lots of questions which she does not want.*”
- “*One day they gave everyone a hot chocolate, but they just gave water to my child.*”

### 3.9. Support with Special Diet by the School

Many parents/caregivers (*n* = 29) positively described the support they received from the school. However, some outlined the amount of work and liaison they have to do with the school team to receive a better service for their children.

Parents/caregivers verbatim quotes:

- “*I have been extremely lucky with the support we have for my son at school. They will do everything they can to ensure my son is as included as we would like him to be. They have gained a lot of knowledge and continue to check in and ask questions or change their ‘usual’ foods where needed.*”
- “*When my daughter has been on residential holidays with the school the staff have been excellent arranging catering with staff wherever they have stayed (France and UK).*”

### 3.10. Negative Comments about School Care for PKU Children

Thematic analysis indicated a further 34 negative experiences with school and management of PKU by respondents. 

Parents/caregivers verbatim quotes:

- “*It took a long time to get an initial meeting and then there was a lot of work over a 3 month period to get everything sorted. There was lots of obstacles and a lot of work and organization at school.*”
- “*Have had to ask for more appointments to see SENCO teacher to discuss issues. She takes very little action.*”

### 3.11. Secondary School Provision

Parents/caregivers gave 10 comments about the issues for children in secondary school. They described the fear children experience and how they do not want to look different from their peers and the difficulties they experience.

Parents/caregivers verbatim quotes:

- “*In a secondary school it is harder to control your child’s diet. You have to try and trust they will do the right thing. You can make a fuss but the children resent you for this.*”
- “*In a secondary school there is no supervision.*”
- “*Although teacher received training it was one teacher out of many- so really not relevant.*”

### 3.12. Administration of Protein Substitute in School

Protein substitute administration was more commonly unsupervised in children in secondary (77%, *n* = 34/44) than primary school (17%, *n* = 11/66) (Pearson Chi-Square Test *p* = 0.001). Those who did not have an IHCP (57%, *n* = 25/44) were less likely to be supervised compared to those who did have a plan (24%, *n* = 18/75) (Pearson Chi-Square test *p* = 0.001). Any supervision was mostly provided by teaching assistants. 

Some parents commented that the school had helped with the transition of protein substitute from a paste to a liquid, others described the measures that the school staff took to ensure that a child took the protein substitute. Some described how they chose not to give protein substitute at school because it was unsupervised and consequently not taken. Others explained there that there was less supervision in secondary school, with one respondent describing a medical room being locked so their child could not gain access to their supply of protein substitute.

Parents/caregivers verbatim quotes:

- “*The school have helped my child with the transition of protein substitute from a paste to a liquid.*”
- “*School returns the empty protein substitute pouch each day to evidence that it has all been taken.*”
- “*The protein substitute is well supervised by teaching assistants. The dietitian and we as parents have spent a lot of time on this.*”
- “*She was telling her teacher that she had drank it when she had not. The teacher just accepted the information from the child. Blood levels went high.*”
- “*We decided to not give my son his substitute at school as this was getting missed.*”

## 4. Discussion

This is the first study to explore the views and experiences of parents and caregivers of children with PKU in school and nursery. Additionally, the care of children with and without an IHCP were studied. The responses to this questionnaire represent approximately 20% of school-aged children with PKU in the UK [9]. The experiences of parents/caregivers in relation to schools were highly variable ranging from excellent support, to care that was unsafe, potentially adversely impacting metabolic control of children with PKU. Findings from this questionnaire suggest that pre-admission school planning, health professional training of school team members, and a carefully written IHCP that is reviewed at least annually are all essential components of successful PKU management within schools. 

Although every child has the right to a varied and nutritious menu in school, uptake of school meals by parents/caregivers of children with PKU was considerably lower than the general population. Only 39% of children with PKU compared with 58% to 79% of UK school aged children received school meals [10]; and 50% of parents did not utilize their child’s entitlement to free school lunches. Some parents/caregivers preferred to give their children packed lunches because of safety concerns, so they could maintain control over their child’s food. Others reported that this allowed their child to retain some anonymity about the condition because a low phenylalanine packed lunch looked like a regular packed lunch. Consequently, this situation further penalizes families with PKU by increasing their workload and expenditure on food when they are already managing a stringent and costly dietary treatment. 

Parents reported numerous barriers to school meals provided by school catering services including poor food quality, inadequate variety, requirement for extra parental organization and liaison, and operational systems in meal delivery (children having to ask for their special meal, wearing lanyards, child photographs) that brought unwelcome attention to the child. When external catering services provided school lunches, greater difficulty with food provision was reported. They appeared ‘rigid’ in their approach using allergy concerns with risks of cross-contamination as reasons for not providing school meals, and refusal to use SLPF’s supplied via parents for children with PKU, despite being unprepared to purchase SLPF’s themselves due to the extra cost and their own operating procedures. This refusal and failure to provide appropriate low phenylalanine school meals is discriminatory [4]. To help children with PKU who are entitled to free school lunches but unable to utilize them, the government should consider issuing money vouchers to assist with extra food costs. 

Around 60% of children with PKU had a written IHCP but it is unknown how this compares with use of IHCPs in other chronic health conditions. There is some data that predates the 2014 education act to suggest that only 50% of children with conditions such as diabetes, epilepsy and asthma had an IHCP [11]. Although IHCP’s are not mandatory, they helped improve care provision for children with PKU at school. Children with PKU with an IHCP were more likely to have protein substitute administration supervised, have alternative suitable low phenylalanine meals prepared, receive supervision at snack and school lunch time and receive feedback from the school staff. It was also evident that some parents worked very hard with schools, particularly at school entry to establish good care for their children. Some described setbacks, but clear management strategies with regular review of the IHCP plan helped. 

IHCP’s should include information about PKU and treatment, including protein substitute (dose, time, administration, storage), snack and meal choices, protein exchanges, and the level of support needed (some secondary school children may be able to take responsibility for their own health needs). It is mandatory that schools ensure that written records are kept of all protein substitute that is administered. If a child is self-managing their protein substitute and low phenylalanine diet within secondary school, this should be clearly stated, with appropriate arrangements for monitoring, documenting who will provide any additional support, and their training needs. There should be a clear pathway with named personnel about how and from whom they can obtain help if issues arise at school. All arrangements should generate confidence for parents and pupils. The Department of Health has also produced IHCP templates which healthcare professionals and schools may find useful [3]. PKU specific templates are also available online from the UK National Society of Phenylketonuria [12]. 

Inadequate staff training and lack of supervision with food was commonly described by parents/caregivers and carried a considerable safety risk for children with PKU. There were several descriptions of children eating or being offered the wrong foods either accidentally or purposely due to inadequate supervision. Better training is needed to enable staff to fully support children at school and this should include all school staff who provide care for children with PKU. Teaching assistants often have an important role in supervising protein substitutes and snacks but are commonly omitted from professional training sessions. Lunch time supervisors are also overlooked for training, but they are central to ensuring that children receive the correct food at mealtimes. Although the parents of a child will often be key in providing relevant information to school staff, training should be provided by a health professional. In addition, availability of online training resources developed by health professionals will help improve the school team’s basic knowledge of PKU. In conditions such as diabetes, it is reported that attitudes of teachers and their lack of understanding impact on their ability to manage the condition [13]. 

Parents/caregivers described some of the school strategies used that led to better management of PKU. Some schools had helped with the transition from a spoonable/paste to a liquid protein substitute. At lunch time, if children were allowed to have a friend queue and visit food counters with them it was considered more discreet and enabled children to feel less special and more supported. Teachers or teaching assistants sitting in the dining room or at the table with the children helped check the correct foods were consumed. Photographing meals pre and post consumption helped parents understand what foods had been offered and eaten by children. Cashless payment systems in secondary schools enabled parents to go online to see what foods their children had purchased. Procedures to cover any transitional arrangements between primary and secondary schools (or nursery and primary school), were also highlighted as important. 

Parents/caregivers commonly described their concerns about social exclusion. Children may be unintentionally excluded because of inadequate inclusive opportunities with suitable food provision. Social exclusion frequently causes psychological harm and can have negative outcomes on emotional and mental health, lowering self-esteem, increasing feelings of anxiety, depression and aggression and may even have a detrimental impact on academic performance [14]. Generally, older children with chronic health conditions are almost three times as likely as healthy peers to suffer social exclusion in school [15], as they are seen as different from their peers [14]. This has previously been reported in PKU [16]. 

The transition into secondary school is naturally associated with greater independence amongst adolescents. Parents reported difficulties with managing a low phenylalanine diet once their child entered secondary school and it was commonly associated with deteriorating blood phenylalanine control [17,18]. Children were self-conscious about their condition and were fearful about mistreatment by peers if their disability became known; dietary management was effectively sacrificed to avoid bullying and harassment by other pupils in school. They commonly avoided any special food that appeared different from regular foods and refused protein substitute administration at school. There was also limited staff training in secondary school, so less teacher empathy and support for the child with PKU. Commonly the position of secondary schools is that children with disability should develop independency with their care needs, but there is a high measure of responsibility on a child as they enter their journey through secondary school. It is important that schools, parents, and school governors work together to help ensure that the secondary school culture is supportive and inclusive and that it encourages acceptance of children with a range of differences. A lack of sensitivity toward people with disabilities is a problem that requires attitude change and training. The impact of children attending secondary school and its association with declining blood phenylalanine control warrants further investigation. 

### Limitations

There are several limitations to this study. This questionnaire was not validated. Data was not collected about individual protein tolerance or about all food provided by school within the day such as breakfast clubs, after school clubs, tuck shops and celebrations in order to ensure that the questionnaire was not too burdensome to complete. The questionnaires were completed at the start of the Covid 19 pandemic, but respondents were asked to document their usual experience at school. Each questionnaire collected information about one child with PKU in a family; it did not refer/collect information about other children in the family with or without PKU. Data was collected based on parents/caregivers’ perception of the service or school incidents, so some answers maybe subjective. The respondents were not randomly selected, and participation was voluntary. Additionally, individuals without internet access may have been unable to participate. The survey was promoted on the NSPKU Twitter and Facebook page, meaning participants were more likely to be NSPKU members who may be more proactive and informed about PKU. Therefore, the survey population may not be representative of the entire PKU population although it is estimated that this questionnaire covers around 20% of the children in school with PKU in the UK.

## 5. Conclusions

There was disparity in the support given to children with PKU across the UK. They received school meals less commonly than their peers, even when they were entitled to ‘free school meals.’ Some catering services discriminated against children with PKU by refusing to provide suitable food; some parents distrusted the school meals service. Children were commonly unsupervised with food, leading to the consumption of inappropriate foods. Improved supervision and communication were associated with a written IHCP. We recommend that every child with PKU should have an IHCP, with mandatory training of all staff involved in their care. It is imperative that every child with PKU is supported in school, and their individual dietary and health needs are met safely and discreetly.

## Figures and Tables

**Table 1:** School type, age group and provision of IHCP.

**School type**	**%**	**Number of children/total number of responses**
State school	92	146/159
Private school	6	10/159
Other (e.g., special needs school)	2	3/159
**Year group in school**	**%**	**Number of children/total number of responses**
Nursery/reception	14	21/154
Primary school Years 1–3 Years 4–6	51 (27) (24)	79/154 (42) (37)
Secondary school Years 7–9 Years 10–11	35 (18) (18)	54/154 (27) (27)
**Provision of Individual Health Care Plan**	**%**	**Number of children/total number of responses**
Yes	60	95/159
No	33	53/159
Don’t know	7	11/159

When considering the provision of written IHCP’s, there was no difference between state or private school or between school year groups (Pearson Chi-Square test, *p* > 0.5).

**Table 2:** Uptake of school lunches and entitlement to free school lunches.

**Numbers of times school lunch is eaten each week prepared by the school**	**%**	**Number of children**
**0**	61	96
**1**	6	10
**2–3**	7	11
**4–5**	26	40
**Total**	100	157
**Entitled to free school lunch**	**%**	**Number of children**
**Yes** **Nursery/Reception** **Primary School** **Secondary School**	35 (71) (40) (19)	56 (15) (31) (10)
**No** **Nursery/Reception** **Primary School** **Secondary School**	64 (29) (60) (81)	96 (6) (47) (43)
**Don’t know**	1	2
**Total**	100	154

**Table 3:** Meal provision within school and type of special low protein foods used.

**School Meal Plans**	**%** **(Number of patients/total number of responses)**
**Food chosen from standard school menu**	32% (*n* = 20/63) *
**Separate low protein meal prepared**	51% (*n* = 32/63)
**Meals provided by parents/caregivers or standard school menu adapted to make it suitable for children with PKU**	17% (*n* = 11/63)
**Common low protein foods substituted used when menus were adapted**	
Low protein pasta (52%, *n* = 33/63) Low protein pizza (48%, *n* = 30/63) Vegan or ‘free from’ low protein cheese (46%, *n* = 29/63) Low protein bread (46%, *n* = 29/63) Low protein ‘meat’/’fish’ substitutes (40%, *n* = 25/63) ‘Fishless’ fingers (17%, *n* = 11/63)	

‘free from’: food without one or more specific ingredients, designed for people with food allergies or other intolerances/diseases). * 40% (*n* = 8/20) of children that had food chosen from standard school menu were taking sapropterin and were permitted a higher protein intake.

**Table 4:** All strategies suggested by parents/caregivers to prevent incorrect foods being eaten by children with PKU in school.

**Supervision at mealtime** Wears lanyard at lunch time so he is recognizable. Other children on special diets also do this so he is not the only one. Poster with his name, picture and instructions on for everyone to see. Not allowed to self-choose food from canteen. Teaching assistant watches her, and she is served based on what we put on her lanyard that she can eat each day. The school have a lunch system where each child’s name is typed into a ticket system which then says which lunch they have based on the parents ordering. He has his own dinner lady on his table that sits with him. No one is allowed to share their lunch.
**Communication/education with school staff** The teacher talks to me before any occasions or food related activity. Teachers know to ring parents to organize if they are doing cookery lessons so products can be provided. They check with me before letting her have anything. They are all very aware and my child has very good awareness himself. Talk to school cook every morning. Have a review meeting every year with the teacher to explain about treatment needs. Regular staff training. Regular update of health care plans. Care plan and pack given by dietitian provide school with information. My child is not allowed to take money to school so she cannot buy food from the tuck shop. Child takes packed lunch. Can only eat from lunch box. Teachers sit at his lunch table. We have a hand over book, if anything off limits was eaten it would be recorded. The teachers and kitchen staff also have the NSPKU booklet, so they know what is allowed and what isn’t. I help the chef with the menus and he runs any new ideas by me. School sends a photo and written comments (and sometimes actual food) to show what has been eaten in a communication book. Breakfast club and after school club use the communication book too.
**Communication with previous school/nursery** School visited the nursery and saw the systems that they had in place there and all the measures that they took which I think helped them visualise them in real terms.

## Data Availability

The data will be made available from the authors upon reasonable request.

## Supplementary Materials

The following is available online at www.mdpi.com/article/10.3390/nu13113863/s1, Full questionnaire.
