# Supplementary material for: Provision and Supervision of Food and Protein Substitute in School for Children with PKU: Parent Experiences
Source: Nutrients. 2021 Oct 28;13(11):3863. doi: 10.3390/nu13113863 (PMC8621748; doi:10.3390/nu13113863)
Supplement: Supplementary file 1 [file nutrients-13-03863-s001.zip › nutrients-1393456-supplementary.pdf]

Supplementary material: Full Questionnaire

Title: Provision of school food for children with PKU: A parent's perspective

1. **Are you a parent / carer of a child under the age of 18 who has PKU?**
   - Yes
   - No

2. **Which type of school does your child currently attend?**
   - State school
   - Private school
   - Boarding school
   - Other

   If you selected other, please specify:

3. **Which year group is your child in?**
   - Nursery/reception
   - Primary school (years 1-3)
   - Primary school (years 4-6)
   - Secondary school (years 7-9)
   - Secondary school (years 10-11)
   - Other

   If you selected other, please specify:

4. **Do you have an Individual Healthcare Plan or a written agreement about how your child's PKU should be managed at school?**
   - Yes
   - No
   - Don't know

5. **Is your child entitled to free school lunches?**
   - Yes
   - No
   - Don't know

6. **How many times per week, if any, does your child normally have lunch provided by the school?**
   - 0
   - 1
   - 2-3
   - 4-5

   What are the reasons for this?

   6b. Would you like your child to have school lunches more often?
   - Yes
   - No

If yes, what are the barriers to this?

7. **Does your child's school provide snacks for children at break time? (e.g. fruit or milk)**
    - Yes
    - No
    - Don't know

    7a.. If yes, is the snack or drink provided suitable for a low protein diet?
    - Yes
    - No

    7b If no, what alternatives are in place for your child?

8. **To what extent are you satisfied or dissatisfied overall with the school lunch service provided for your child?**
   Please tick:
    - Very satisfied
    - Fairly satisfied
    - Neither satisfied nor dissatisfied
    - Very dissatisfied

9. **For your child, are meals chosen from the same menu as the other children choose from, or are specific alternative meals made?**
    - Meals are chosen from the same menu as the other children
    - Specific alternative meals are made
    - Other

    If you selected other, please specify:

10. **Which of the following are included in the school lunch service at your child's school? Please tick all that apply.**
    - Low protein bread
    - Low protein pizza bases
    - Low protein sausage/burger/fish mix
    - Low protein pasta
    - Low protein flour
    - Low protein cake mix
    - Low protein chocolate
    - Quorn Fishless fingers
    - Low protein cheese
    - Low protein milk
    - Low protein meal alternatives that have been prepared at the parents at home and need reheating
    - None of the above

    10.a What are the reasons given for this?

    10.b Who provides the following low protein food?
    - Low protein bread (Hospital/ Parents/ School catering/ Not applicable)
    - Low protein pizza bases (Hospital/ Parents/ School catering/ Not applicable)

- Low protein sausage/burger/fish mix (Hospital/ Parents/ School catering/ Not applicable)
- Low protein pasta (Hospital/ Parents/ School catering/ Not applicable)
- Low protein flour (Hospital/ Parents/ School catering/ Not applicable)
- Low protein cake mix (Hospital/ Parents/ School catering/ Not applicable)
- Low protein chocolate (Hospital/ Parents/ School catering/ Not applicable)
- Quorn Fishless fingers (Hospital/ Parents/ School catering/ Not applicable)
- Low protein cheese (Hospital/ Parents/ School catering/ Not applicable)
- Low protein milk (Hospital/ Parents/ School catering/ Not applicable)
- Low protein meal alternatives that have been prepared at the parents at home (Hospital/ Parents/ School catering/ Not applicable) and need reheating
- None of the above (Hospital/ Parents/ School catering/ Not applicable)

11. Do catering staff at your child's school measure/weigh exchange to meet your child's exchange allowance? (e.g. mashed potato)

- Yes
- No
- Don't know

If no, what are the reasons given for this?

12. Have the teachers at your child's school received training from a health professional about PKU and a low phenylalanine diet?

- Yes
- No
- Don't know

12.a. If yes, how long was this?

- Less than 12 months ago
- 1-2 years ago
- 3-4 years ago
- Over 4 years ago

12.b. If yes, who conducted the training?

- Child's dietitian in person
- Child's dietitian by letter/information pack
- Child's nurse
- Hospital support worker
- Other

If you selected other, please specify:

13. Have the catering team at your child's school received training from a health professional about PKU and a low phenylalanine diet?

- Yes
- No
- Don't know

13.a If yes, how long ago was this?

- Less than 12 months ago
- 1-2 years ago

- 2-3 years ago
- Over 3 years ago

  13.b. If yes, who conducted training?
- Child's dietitian in person
- Child's dietitian by letter/information pack
- Child's nurse
- Hospital support worker
- Other

  13.b.i. If you selected other, please specify:

14. Using the scale below, rate how knowledgeable you feel the staff in your child's school are about the dietary requirements of children with PKU?

- Teachers (no knowledge/ very limited knowledge/ neither knowledgeable nor unknowledgeable/ fairly knowledgeable/ extremely knowledgeable/ Not applicable)
- Teaching assistants (no knowledge/ very limited knowledge/ neither knowledgeable nor unknowledgeable/ fairly knowledgeable/ extremely knowledgeable/ Not applicable)
- School cooks (no knowledge/ very limited knowledge/ neither knowledgeable nor unknowledgeable/ fairly knowledgeable/ extremely knowledgeable/ Not applicable)
- Lunch time supervisors (no knowledge/ very limited knowledge/ neither knowledgeable nor unknowledgeable/ fairly knowledgeable/ extremely knowledgeable/ Not applicable)

15. **Who supervises your child eating the following?**

- Lunch (Teacher/ Teaching assistant/ Catering staff/ Lunchtime supervisor/ Unsupervised/ Don't know/ Other)

  - If you selected other, please specify:
- Snack (Teacher/ Teaching assistant/ Catering staff/ Lunchtime supervisor/ Unsupervised/ Don't know/ Other)

  - If you selected Other, please specify:
- Protein substitute (Teacher/ Teaching assistant/ Catering staff/ Lunchtime supervisor/ Unsupervised/ Don't know/ Other)

  - If you selected other, please specify:

16. Do you receive feedback in any format about what has been eaten by your child at school?

- Yes
- No

16.a If yes, what format do you receive this in?

- Verbal feedback
- Written record
- Photographic record
- Online report
- Other

If you selected other, please specify:

16.b If yes, does this include feedback about protein substitute?
- Yes
- No
- Not applicable

17. To your knowledge, has your child ever eaten foods which are not permitted as part of their PKU diet while at school?
- Yes
- No
- Don't know
-

17.a If yes, how many times had this happened in the last 6 months?
- 1
- 2-3
- 4-5
- Greater than 5

17.b Please give more details of what happened (e.g. where did it happen, what was eaten):

17.c Do you feel that you were adequately informed of the incident?
- Yes
- No

17.c.i If no, what do you feel could have been done better?

17.d Are there strategies in place at your child's school to avoid incidents like this happening?
- Yes
- No
- Don't know

17.d.i If yes, what are these?

18. Please use the box below to make any additional comments about provision or supervision of food or protein substitute at your child's school.
